# Associations between teamwork and implementation outcomes in multidisciplinary cross-sector teams implementing a mental health screening and referral protocol

**DOI:** 10.1186/s43058-023-00393-8

**Published:** 2023-02-10

**Authors:** Elizabeth A. McGuier, Gregory A. Aarons, Kara A. Byrne, Kristine A. Campbell, Brooks Keeshin, Scott D. Rothenberger, Laurie R. Weingart, Eduardo Salas, David J. Kolko

**Affiliations:** 1 Department of Psychiatry, School of Medicine, University of Pittsburgh, 3811 O’Hara Street, Pittsburgh, PA 15213 USA; 2 Department of Psychiatry, University of California-San Diego, San Diego, CA USA; 3 ACTRI Dissemination and Implementation Science Center, University of California-San Diego, San Diego, USA; 4 Child and Adolescent Services Research Center, San Diego, CA USA; 5 David Eccles School of Business, Kem C. Gardner Policy Institute, University of Utah, Salt Lake City, UT USA; 6 Center for Safe and Healthy Families, Department of Pediatrics, University of Utah, Salt Lake City, UT USA; 7 Department of Medicine, University of Pittsburgh School of Medicine, Pittsburgh, PA USA; 8 Tepper School of Business, Carnegie Mellon University, Pittsburgh, PA USA; 9 Rice University, Houston, TX USA

**Keywords:** Team, Teamwork, Implementation outcomes, Mental health screening, Child maltreatment

## Abstract

**Purpose:**

Teams play a central role in the implementation of new practices in settings providing team-based care. However, the implementation science literature has paid little attention to potentially important team-level constructs. Aspects of teamwork, including team interdependence, team functioning, and team performance, may affect implementation processes and outcomes. This cross-sectional study tests associations between teamwork and implementation antecedents and outcomes in a statewide initiative to implement a standardized mental health screening/referral protocol in Child Advocacy Centers (CACs).

**Methods:**

Multidisciplinary team members (*N* = 433) from 21 CACs completed measures of team interdependence; affective, behavioral, and cognitive team functioning; and team performance. Team members also rated the acceptability, appropriateness, and feasibility of the screening/referral protocol and implementation climate. The implementation outcomes of days to adoption and reach were independently assessed with administrative data. Associations between team constructs and implementation antecedents and outcomes were tested with linear mixed models and regression analyses.

**Results:**

Team task interdependence was positively associated with implementation climate and reach, and outcome interdependence was negatively correlated with days to adoption. Task and outcome interdependence were not associated with acceptability, appropriateness, or feasibility of the screening/referral protocol. Affective team functioning (i.e., greater liking, trust, and respect) was associated with greater acceptability, appropriateness, and feasibility. Behavioral and cognitive team functioning were not associated with any implementation outcomes in multivariable models. Team performance was positively associated with acceptability, appropriateness, feasibility, and implementation climate; performance was not associated with days to adoption or reach.

**Conclusions:**

We found associations of team interdependence, functioning, and performance with both individual- and center-level implementation outcomes. Implementation strategies targeting teamwork, especially task interdependence, affective functioning, and performance, may contribute to improving implementation outcomes in team-based service settings.

**Supplementary Information:**

The online version contains supplementary material available at 10.1186/s43058-023-00393-8.

Contributions to the literature

• This study raises awareness of teams and team processes as important areas from organizational science that can be applied to implementation research.
• Our findings provide evidence of meaningful associations between teamwork in multidisciplinary cross-sector teams and implementation antecedents and outcomes.
• Aspects of team interdependence, functioning, and performance were associated with implementation antecedents and outcomes in a multidisciplinary team-based setting.
• Understanding how teamwork affects implementation antecedents and outcomes can improve efforts to implement new practices in team-based settings.

## Background

Team-based care is increasingly common in healthcare and human service settings [1–7]. Team-based care is driven by the increasing complexity of modern healthcare and reflects a shift away from the sole provider model to purposeful inclusion of multiple professionals with varying skills and expertise [6, 8]. In healthcare, team-based care is defined as “the provision of health services…by at least two health providers who work collaboratively with patients and their caregivers…to accomplish shared goals within and across settings to achieve coordinated, high-quality care” [6]. In team-based service settings, team members share responsibility for outcomes and depend on one another to complete their work.

Implementation of evidence-based practices in team-based settings requires teams to work together to respond to new demands and changing expectations. Accordingly, teamwork is likely to affect the implementation of new practices in settings that provide team-based care. We use the term “teamwork” to refer to a broad array of team constructs, including team structure (e.g., size, composition), processes and emergent states (e.g., communication, cohesion), and team performance/effectiveness. Although much is known about implementation barriers, facilitators, and strategies at the individual and organizational levels, less is known about how team constructs are associated with implementation processes and outcomes [9, 10].

Most implementation theories, models, and frameworks do not explicitly highlight the team level or include team constructs as determinants. In the Exploration, Preparation, Implementation, Sustainment (EPIS) framework [11, 12], teams can be conceptualized as part of the inner context alongside individual and organizational characteristics, within the outer context, and as bridging factors when teams consist of coalitions or collaborators that cross systems and organizations [13, 14]. In addition, the oft-overlooked “interconnections and linkages” construct in EPIS clearly implicates how teams within and across outer and inner contexts should be considered and used in the development of team-based implementation strategies [12].

Several studies in diverse settings have found that aspects of teamwork are associated with implementation outcomes. In healthcare clinics, Lukas and colleagues found that greater team knowledge and skills and team participation were associated with greater implementation of key changes to improve access to care [15, 16]. They suggested that successful teams were those that seek information, use data, assess their progress, and learn from others [15, 16]. In teams implementing dialectical behavior therapy, greater team cohesion, communication, and climate for innovation were associated with the implementation of more program elements [17]. Another study found that information sharing and learning within teams was associated with better implementation of educational reforms [18].

Teamwork is also associated with greater implementation progress over time. Cramm and colleagues studied the implementation of child-to-adult healthcare transition programs for adolescents with chronic health conditions by 29 teams in hospitals and rehabilitation units. More positive team climate (i.e., shared vision, participative safety, task orientation, support for innovation) at the study start was associated with greater improvements in the quality of chronic care delivery 1 year later [19]. In addition, changes in team climate during the 1-year period were associated with greater improvement in care delivery (i.e., movement toward optimal chronic care delivery) [19]. These findings were consistent across teams working with different patient populations (e.g., diabetes, cystic fibrosis, neuromuscular disorders), suggesting that the influence of team climate generalizes across teams and settings [19]. Another study found that primary care practices reporting better teamwork were more likely to be in later stages of transformation to patient-centered medical homes than practices with poorer teamwork [20]. Finally, team functioning, assessed with a composite of measures, has been positively associated with the sustainment of trauma-focused evidence-based practices in outpatient mental health clinics [21].

Support for the hypothesis that teamwork influences implementation also comes from studies of “implementation teams”—i.e., teams created to lead implementation efforts [22]. One study found that teams with better teamwork were more likely to be early adopters [23]. Another study found that implementation team members’ perceptions of effectiveness were associated with the number and depth of changes made, based on data from multiple sources, when implementing chronic care models [24].

Overall, there is some evidence indicating that teamwork matters for implementation. However, the existing literature has relied primarily on broad measures of team constructs, with little consistency across studies. In addition, limited efforts have been made to connect this work with the substantial organizational theory and research on teams and teamwork. Advancing our understanding of team-level influences on implementation requires greater specificity, depth, and rigor in our conceptualization, measurement, and interpretation of research on teams and implementation.

### Conceptualizing teamwork in relation to implementation outcomes

The input-mediator-outcome (IMO) framework differentiates between team inputs (i.e., features of team members, teams, and their context), mediators (i.e., team processes and emergent states that transform inputs into outcomes), and outcomes (i.e., valued results of team activities) [25–28]. It also acknowledges that teams are affected by complex temporal dynamics and situated within organizational and system contexts [25, 28–31].

**Fig. 1:**
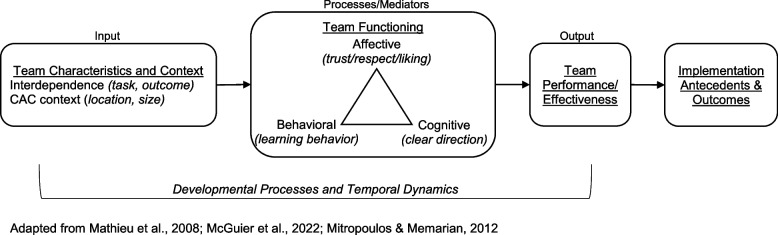
Input-mediator-outcome framework of team effectiveness

For this study, we selected team constructs based on the IMO framework, illustrated in Fig. 1. We focus on team interdependence, team functioning (i.e., processes and states), and team performance [32–34], described in more detail below. Although closely intertwined, specific dimensions of teamwork may influence implementation differently. Applying well-established theoretical models of teams and distinguishing between team constructs can advance our understanding of how teams influence implementation outcomes.

Interdependence is a structural property of teams that influences how team members work together [32]. It comes in two forms—task interdependence and outcome interdependence [32]. Task interdependence is the extent to which the team’s work requires exchanges of resources and coordinated workflows, and outcome interdependence is the extent to which outcomes are measured and rewarded at the team (vs. individual) level [32, 35]. Teams with greater task and outcome interdependence tend to engage in more collaboration and cooperation, which has been shown to facilitate change [32, 36]. More task interdependence has been associated with greater innovation in heterogeneous teams [37], and a meta-analysis found that outcome interdependence is positively associated with innovation in work teams [38].

Team functioning includes processes and emergent states reflecting what team members think, feel, and do. Team processes and states may be affective (e.g., respect), behavioral (e.g., communication), or cognitive (e.g., shared goals) [33, 34]. Decades of research in diverse work settings have shown that affective, behavioral, and cognitive team functioning are positively associated with team performance [32–35, 39–44]. In healthcare settings, specifically, better team functioning is associated with better service quality, improved patient safety, and better clinical outcomes for those served by the team [7, 45, 46]. Aspects of team functioning associated with effective implementation include affective states (e.g., liking team members, feeling like a team, psychological safety), behavioral processes (e.g., communication, learning behavior, support for innovation), and cognitive states (e.g., shared vision and goals, team knowledge) [15–19].

For this study, we focus on affective integration (i.e., liking, trust, and respect within the team), learning behavior (i.e., extent to which team members seek feedback, share information, and reflect on their performance), and clear shared direction (i.e., agreement on vision and goals). Learning behavior should directly impact the team’s ability to adapt to changes such as the adoption of a new practice [47–49]. Affective functioning is associated with the extent of learning behavior in teams, with teams experiencing more positive affective states engaging in more learning behavior [50]. Shared vision and goals within teams are associated with innovation [38] and overall performance [41, 51].

Lastly, team performance or effectiveness refers to team-level productivity, efficiency, and the quality of the team’s work. It is multidimensional, context-specific, and can be measured objectively and/or subjectively [27, 28, 52]. In team-based settings, team members’ perceptions of overall performance are likely to be associated with implementation outcomes, as better-performing teams may be more able to make the changes needed to implement a new practice [20, 21, 23, 24].

Teamwork may affect both implementation antecedents and implementation outcomes, differentiated in the Consolidated Framework for Implementation Research (CFIR) outcomes addendum [53]. Implementation outcomes are posited to be indicators that reflect the actual success or failure of the implementation effort (e.g., adoption, fidelity), while antecedents are constructs that predict implementation outcomes (e.g., readiness). For this study, antecedents include the acceptability, appropriateness, and feasibility of the innovation, as well as implementation climate [53]. Implementation outcomes include adoption and reach [53, 54].

## Current study

The current study examined associations between multiple dimensions of teamwork and implementation antecedents and outcomes during a statewide initiative to implement a standardized mental health screening/referral protocol, the Care Process Model for Pediatric Traumatic Stress (CPM-PTS), in Child Advocacy Centers. Child Advocacy Centers (CACs) provide interagency responses to allegations of child sexual abuse and other maltreatment and rely on multidisciplinary team members employed by independent organizations (e.g., law enforcement, child welfare, prosecution, medicine, mental health, victim advocacy) [3, 55]. Team membership and boundaries are fluid and dynamic [56, 57]. This setting provides an excellent opportunity to examine how teamwork is associated with the implementation of new practices.

The CPM-PTS is a standardized protocol for identifying and responding to symptoms of traumatic stress and suicidality in children following allegations of maltreatment [58, 59]. It provides frontline staff with evidence-based screening tools, structured clinical pathways, and technology-guided decision support to enhance screening and referral efforts. Screening and referral protocols such as the CPM-PTS aim to improve recognition of mental health needs, reduce variability and inefficient use of resources, and facilitate engagement in treatment [60, 61].

We assessed multidisciplinary team interdependence, functioning, and performance as well as implementation antecedents with a cross-sectional survey. We collected data on implementation outcomes during the first 2 years of implementation. We hypothesized that greater team interdependence, more adaptive team functioning, and better team performance would be associated with more positive antecedents and better implementation outcomes.

## Methods

### Statewide implementation of the CPM-PTS in Child Advocacy Centers

All 24 CACs in a single state in the USA were invited to implement the CPM-PTS. Most CACs were in rural or frontier counties (10 rural, 8 frontier), with 6 CACs in urban counties [62]. There were 4 CACs, all in frontier counties (< 7 people per square mile), that were affiliated with another CAC (i.e., satellite sites). During the first 2 years of implementation (April 2018 through March 2020), staff from 22 of 24 CACs completed training, and 19 CACs began using the CPM-PTS with children and families. Byrne and colleagues [58] described the development and implementation of the CPM-PTS and identified facilitators and barriers to its use.

### Survey participants and procedures

We conducted an anonymous online survey of CAC multidisciplinary team members [44]. Each CAC has one associated multidisciplinary team. Survey invitations were emailed to all CAC directors with a request that they forward the invitation to their team members. All team members identified by directors were eligible to participate. Because CACs implemented the CPM-PTS at different times during the 2-year period, the survey occurred 1–18 months after training in the CPM-PTS. Participants received a $5 Amazon.com gift card for completing the survey. All participating CACs received a summary of their results, and CACs with > 75% participation received $150. All procedures were approved by the University of Utah Institutional Review Board.

### CAC characteristics

We assessed CAC characteristics that may be associated with implementation outcomes. CACs were classified by location (rural/frontier vs. urban) [62] and as independent or satellite sites. Other characteristics of interest were team size at the time of the survey (director report), the average number of children served each month (2019 administrative data), and survey participation rate.

### Team measures

#### Team interdependence

Task interdependence (i.e., extent to which the team’s work requires exchange of resources and coordinated workflows; 5 items) and outcome interdependence (i.e., extent to which outcomes are measured and rewarded at the team level; 2 items) were assessed with items from van der Vegt and colleagues [35]. Participants rated their agreement with each item on a 5-point Likert scale (1 “strongly disagree” to 5 “strongly agree”). Internal consistency reliability for both scales was good (task interdependence *α* = 0.79; outcome interdependence *α* = 0.78).

#### Team functioning

The survey included established measures of affective, behavioral, and cognitive team processes and states, specifically affective integration, learning behavior, and clear shared direction. Affective integration (i.e., extent to which team members like, trust, and respect one another) was assessed with 11 items [63]. Participants rated each item on a 5-point Likert scale (1 “strongly disagree” to 5 “strongly agree”). Confirmatory factor analysis supports a single factor structure for these items [63], and the scale had good reliability in this study (*α* = 0.91). Learning behavior (i.e., how much the team tries to keep learning and improving) was assessed with 7 items (*α* = 0.75), and direction (i.e., extent to which team members understand the team’s purpose and goals) was assessed with 3 items (*α* = 0.90) [50]. For both learning behavior and direction, participants rated each statement on a 7-point Likert scale (1 “very inaccurate” to 7 “very accurate”). Psychometric research has found good internal consistency reliability and discriminant validity for these scales [50]. For all three scales, prior research supports their validity and use in measuring team-level constructs [50, 63].

#### Team performance

The overall quality of work done by the team was assessed with 5 items (e.g., “The quality of work done by this team is improving over time”) [50]. Participants rated items on a 7-point Likert scale (1 “very inaccurate” to 7 “very accurate”). Prior research found that this scale had strong construct validity, internal consistency reliability, and team-level convergence [50]. Internal consistency reliability in this study was good (*α* = 0.77).

### Dependent variables: implementation antecedents

#### Acceptability, appropriateness, and feasibility of the CPM-PTS

Participants were provided a brief description of the CPM-PTS and rated its acceptability (4 items), appropriateness (4 items), and feasibility (4 items) on a 5-point scale from 1 “completely disagree” to 5 “completely agree” [64]. Internal consistency reliability for these scales was excellent (*α* = .95–.99). Because items were individually referenced (e.g., “I like the CPM-PTS”), these outcomes were considered individual-level implementation antecedents.

#### Implementation climate

Implementation climate for the CPM-PTS (i.e., extent to which use is expected, supported, and recognized) was assessed with 4 items from Fernandez and colleagues [65, 66]. These items were only completed by participants who reported that their site was using or had previously used the CPM-PTS (*n* = 186; 48%). Participants rated their agreement with each item on a 5-point Likert scale (1 “strongly disagree” to 5 “strongly agree”). Internal consistency was good (*α* = 0.78). All items were group-referenced (e.g., “Team members are expected to help the CAC meet its goals related to the CPM-PTS”), and implementation climate was considered a center-level implementation antecedent [66].

### Dependent variables: implementation outcomes

#### Days to adoption

Adoption was indicated by whether the CAC ever administered the CPM-PTS (i.e., any screening record entered in the electronic system). For CACs that adopted the CPM-PTS, we calculated time to adoption as the number of days from initial training until the first use of the CPM-PTS (i.e., date of first completed screening).

#### Reach

We assessed the reach of the CPM-PTS in each CAC by using administrative data to calculate quarterly screening rates (i.e., completed screenings/eligible children during 3-month periods) (see 58 for more details). We report screening rates for the first quarter following training as well as the average screening rate for all quarters following training. Because the timing of implementation varied by CAC, the number of quarters with screening data ranged from 2 to 8 quarters (*M* = 6.2 quarters).

### Statistical analyses

Our outcomes of interest included both individual-level (i.e., acceptability, appropriateness, feasibility) and center-level (i.e., implementation climate) implementation antecedents as well as center-level implementation outcomes (i.e., days to adoption, screening rates). Prior to conducting analyses of center-level variables, we assessed within-team agreement on measures of team interdependence, functioning, and performance, as well as implementation climate. We used the average deviation index (AD) to determine if measures could be justifiably aggregated to the team level [67–69]. The AD_md_ index quantifies the average deviation from the median and is more sensitive and robust than the average deviation of the mean (AD_M_) [67, 68]. It is preferable to *r*_*wg*_ because it provides an estimate of within-team agreement in the metric of the original response scale and does not require assumptions about the distribution of expected null responses [67]. For each survey measure, the median AD_M_ value across teams was below the recommended upper limit [68], indicating sufficient agreement within teams to justify aggregation. Therefore, we created aggregate scores for each CAC reflecting the median of team member scores.

Our first set of analyses tested associations between CAC characteristics and implementation antecedents and outcomes. Mixed models analyses were conducted in R using the *nlme* package and restricted maximum likelihood estimation (REML); other analyses were conducted in SPSS. We constructed multivariable linear mixed models to test the contribution of team size, location (rural/frontier vs. urban), and participation rate to individual-level antecedents (i.e., acceptability, appropriateness, feasibility). For center-level antecedents and outcomes, we estimated Pearson correlations to test if number of children served, team size, and participation were associated with outcomes, and we conducted independent samples *t*-tests to test the differences between rural/frontier and urban locations and between independent and satellite CACs.

Next, we tested the hypothesized associations between team constructs and implementation antecedents and outcomes. Each outcome was examined separately. The first set of analyses for an outcome included measures of task and outcome interdependence. The second set of analyses included measures of affective, behavioral, and cognitive functioning. The final set of analyses included the team performance measure. For all analyses, we assumed a type 1 error rate of *α* = 0.05 and made no adjustments for multiplicity.

For individual-level outcomes (i.e., acceptability, appropriateness, and feasibility), we constructed linear mixed models including a random effect to account for clustering within CACs. Only cases with complete data were included. For center-level outcomes, we first plotted the associations and estimated Pearson correlations between aggregated team measures and outcomes. Then, we constructed multivariable regression models to test the significance of associations. We used Cox regression models for the days to adoption outcome and linear regression models for other outcomes.

## Results

### CAC characteristics, participation rates, and participant background

Team members from 21 CACs participated in the survey. The director of the remaining 3 CACs did not respond to repeated invitations; these 3 non-responding CACs served < 1% of cases in the state. CACs varied in team size (range = 9–110 members; *M* = 29^1^) and the average number of children served (range = 3﻿–94 per month; *M* = 20). A total of 433 team members participated in the survey. Most teams had high participation rates, with more than 75% participation at 14 CACs (range 33–100%; *M* = 78%). Across all teams, we estimated that 72% of individual team members invited to participate in the survey did so.

Participants represented disciplines typically involved in CAC multidisciplinary teams, including law enforcement (33%), child protective services (19%), prosecution (10%), victim advocacy (9%), CAC administration (7%), mental health (6%), medicine (4%), and others (e.g., probation, forensic interviewing; 13%). Participants identified as non-Hispanic white (90%), Hispanic/Latinx (8%), or belonging to other racial/ethnic groups (2%) and as female (56%), male (43%), or nonbinary or not specified (1%). Participant age varied; the most common category was 36–45 years of age (36%). Most participants (83%) had been part of their CAC team for more than 1 year.

Analyses of center-level outcomes include data from 19 CACs that adopted the CPM-PTS (14 rural/frontier, 5 urban). Two CACs that participated in the survey did not adopt the CPM-PTS. Days to adoption and reach data were available for 16 CACs; two CACs in the same county that combined record-keeping are counted as one CAC for these outcomes only. There was no other team-/center-level missing data. Days to adoption ranged from 0 to 127 (*M* = 37; SD = 38) and were positively skewed. The first quarter after training had the lowest average screening rate across CACs (*M* = 39%; SD = 34%; range = 0–100%); average screening rates for later quarters were relatively stable. The average screening rate for all quarters following training ranged from 10 to 100% (*M* = 53%; SD = 24%). There was considerable variability in screening rates both within and across CACs [58].

### Associations between CAC characteristics, survey participation, and outcomes

Rural/frontier CACs served fewer children (*M* = 9.54 [SD = 6.32] vs. *M* = 48.10 [SD = 31.23] children/month) and had smaller teams (*M* = 20.93 [SD = 9.06] vs. *M* = 50.4 [SD = 34.67]) than urban CACs. Team size and number of children served were strongly correlated (*r* = .87). The correlation between team size and survey participation was significant (*r* = − .54, *p* < .05); larger teams had lower participation rates.

CAC location, team size, and survey participation rates were not associated with any individual-level outcomes in multilevel models. There were no significant differences in survey participation or center-level outcomes between rural/frontier and urban CACs or between independent and satellite CACs. Although there was a trend for CACs that served fewer children to have higher average screening rates (*r* = − .43, *p* = .09), CAC characteristics and survey participation rates were not significantly associated with any center-level implementation antecedents or outcomes. Because team size was strongly correlated with number of children served and survey participation, we considered team size as a possible covariate in our analyses of center-level outcomes. We also considered the number of quarters with screening data as a possible covariate in models testing associations with screening rates.

### Associations with individual-level implementation antecedents: acceptability, appropriateness, and feasibility

Linear mixed models of individual-level outcomes included 384 team members with complete data (89%). The results are shown in Table 1. Descriptive data and correlations for individual-level measures are shown in Additional file 1. Task and outcome interdependence were not significantly associated with acceptability, appropriateness, or feasibility of the CPM-PTS. In the team functioning model, affective integration was significantly positively associated with acceptability, appropriateness, and feasibility; learning behavior and direction were not associated with any outcome. Lastly, team member-rated performance was significantly positively associated with acceptability, appropriateness, and feasibility.

**Table 1:** Individual-level implementation outcomes: results of linear mixed models

	***Acceptability***		***Appropriateness***		***Feasibility***	
	**Unstandardized coefficient (SE)**	**95% CI**	**Unstandardized coefficient (SE)**	**95% CI**	**Unstandardized coefficient (SE)**	**95% CI**
*Team interdependence model*						
Task interdependence	0.08 (0.05)	− 0.02–0.19	0.05 (0.06)	− 0.06–0.16	0.07 (0.05)	− 0.04–0.18
Outcome interdependence	0.01 (0.04)	− 0.07–0.10	0.03 (0.05)	− 0.06–0.13	0.04 (0.05)	− 0.05–0.13
*Team functioning model*						
Affective integration	0.26 (0.08)^**^	0.10–0.43	0.24 (0.09)^**^	0.07–0.41	0.25 (0.08)^**^	0.09–0.42
Learning behavior	0.02 (0.06)	− 0.10–0.13	− 0.03 (0.06)	− 0.15–0.09	0.03 (0.06)	− 0.08–0.15
Clear direction	− 0.03 (0.04)	− 0.12–0.06	0.01 (0.05)	− 0.08–0.10	− 0.03 (0.04)	− 0.12–0.06
*Team performance model*						
Team member-rated performance	0.10 (0.04)^*^	0.02–0.18	0.10 (0.04)^*^	0.01–0.18	0.09 (0.04)^*^	0.01–0.17

Conditional intraclass correlation coefficients (ICCs) for all models were < 0.001

*CI* Confidence interval

^**^p < 0.01

^*^p < 0.05

### Associations with center-level implementation antecedents and outcomes: implementation climate, days to adoption, and screening rates

Plots depicting associations between team measures and center-level implementation antecedents and outcomes are provided in Additional file 2. Descriptive data and correlations are shown in Table 2, and the results of the regression analyses are shown in Table 3. For regression analyses, the patterns of findings were the same with and without covariates; we present the unadjusted results.

**Table 2:** Descriptive statistics and correlations among aggregated team measures and center-level outcomes

	**M (SD)**	**Range**	**2**	**3**	**4**	**5**	**6**	**7**	**8**	**9**	**10**
1. Task interdependence	3.95 (.20)	3.60–4.30	.20	.29	.18	.29	.49^*^	.67^**^	− .32	.60^*^	.55^*^
2. Outcome interdependence	3.88 (.24)	3.50–4.25		.45^+^	.70^**^	.69^**^	.58^*^	.44^+^	− .52^*^	.01	− .12
3. Affective integration	4.27 (.25)	3.82–4.82			.60^**^	.55^*^	.70^**^	.53^*^	− .05	− .20	.07
4. Learning behavior	4.59 (.22)	4.14–5.00				.76^**^	.64^**^	.48^*^	− .38	.03	− .03
5. Clear direction	5.53 (.54)	4.00–6.67					.73^**^	.55^*^	− .41	.04	− .00
6. Team member-rated performance	5.99 (.34)	5.30–6.50						.71^**^	− .32	.18	.34
7. Implementation climate	3.74 (.28)	3.13–4.25							− .27	.42	.65^**^
8. Days to adoption^1^	37.25 (38.46)	0–127								− .59^*^	− .21
9. First quarter screening rate^1^	39% (34%)	0–100%									.76^**^
10. Average screening rate^1^	53% (24%)	10–100%									

*N* = 19 teams

^**^p < .01

^*^p < .05

^+^*p* < .10

^1^*n* = 16 teams

**Table 3:** Center-level implementation outcomes: results of regression models

	*Days to adoption* (*n* = 16)	*Implementation climate* (*n* = 19)	*First quarter screening rate* (*n* = 16)	*Average screening rate* (*n* = 16)
	*χ*^*2*^* for change from the null model* Hazard ratio95% CI	*F for change in R*^*2*^* from the null model R*^*2*^* (adjusted R*^*2*^*)* *B *(SE)95% CI		
*Team interdependence model*	*3.67*	9.62^*^ *0.55 (0.49)*	*3.80*^+^ *0.37 (0.27)*	*3.67*^+^ *0.36 (0.26)*
Task interdependence	3.74 0.16–87.60	0.87 (0.25)^**^ 0.35–1.39	1.12 (0.41)^*^ 0.24–2.00	0.78 (0.30)^*^ 0.15–1.42
Outcome interdependence	4.59 0.52–40.25	0.37 (0.20)^+^ − 0.06–0.80	− 0.15 (0.29) − 0.78–0.48	− 0.23 (0.21) − 0.69–0.23
*Team functioning model*	*2.79*	*2.99+* *0.38 (0.25)*	*0.33* *0.08 (− 0.16)*	*0.05* *0.01 (− 0.24)*
Affective integration	0.31 0.03–3.13	0.37 (0.29) − 0.26–0.99	− 0.43 (0.44) − 1.38–0.52	0.11 (0.33) − 0.60–0.82
Learning behavior	3.30 0.01–937.75	0.04 (0.42) − 0.86–0.94	0.27 (0.85) − 1.58–2.11	− 0.16 (0.63) − 1.54–1.22
Clear direction	2.06 0.29–14.68	0.18 (0.17) − 0.18–0.54	0.05 (0.29) − 0.58–0.67	0.02 (0.21) − 0.45–0.48
*Team performance model*	*1.13*	17.47^**^ *0.51 (0.48)*	*0.45* *0.03 (− 0.04)*	*1.78* *0.11 (0.05)*
Team member-rated performance	2.59 0.45–15.04	0.59 (0.14)^**^ 0.29–0.89	0.19 (0.28) − 0.42–0.79	0.26 (0.20) − 0.16–0.68

*CI* confidence interval

^**^p < 0.01

^*^p < 0.05

^+^*p* < 0.10

Task interdependence was significantly positively correlated with implementation climate, first-quarter screening rate, and average screening rate. Outcome interdependence was marginally positively correlated with implementation climate and significantly negatively correlated with days to adoption. Findings were similar in regression models including both task and outcome interdependence. Task interdependence was significantly positively associated with implementation climate, first-quarter screening rate, and average screening rate, and outcome interdependence was marginally associated with implementation climate.

All three measures of team functioning (i.e., affective integration, learning behavior, clear direction) were significantly positively correlated with implementation climate. They were not significantly associated with any other center-level outcome, although correlations of both learning behavior and clear direction with days to adoption were relatively large (*r* > − .35). In multivariable regression models, team functioning measures were not significantly associated with any outcome.

Team performance was strongly positively correlated with implementation climate. Performance was not significantly correlated with other outcomes, although correlations with days to adoption and average screening rate were in hypothesized directions. Regression findings were similar; team performance was significantly positively associated with implementation climate and not associated with any other center-level outcomes.

## Discussion

This study examined associations of team interdependence, functioning, and performance with implementation outcomes in a statewide sample of Child Advocacy Center multidisciplinary teams implementing the CPM-PTS, a mental health screening/referral protocol. The teams included in this study were generally well-performing, and implementation antecedents (i.e., acceptability, appropriateness, feasibility, implementation climate) were moderately positive. There was considerable variability in implementation outcomes, specifically days to adoption and screening rates, across centers. We found high levels of within-team agreement on implementation climate, indicating shared perceptions of implementation climate within these cross-agency teams. Prior research has examined implementation climate at the individual and organizational levels [66]; our findings extend this work by demonstrating that implementation climate can be measured at the team level in team-based service settings. Measures of team interdependence, functioning, and performance were all positively associated with implementation climate, and stronger implementation climate was associated with higher average screening rates. Future research should further examine how team constructs may contribute to the development and maintenance of implementation climate in team-based settings.

Neither task nor outcome interdependence was associated with team members’ perceptions of the CPM-PTS. Greater task interdependence was associated with more positive implementation climate and higher screening rates. Greater outcome interdependence was correlated with fewer days to adoption, but this association was no longer significant in a multivariable Cox regression model. These findings suggest that teams with greater reliance on one another to share resources and coordinate workflows may be better able to make the changes needed to consistently use the CPM-PTS.

Team members who reported greater affective integration (i.e., liking, trust, and respect) within their team had more positive attitudes toward the CPM-PTS, and at the team level, affective integration was positively correlated with implementation climate. However, affective functioning was no longer significantly associated with implementation climate in multivariable models, and it was not associated with center-level time to adoption or reach. It is possible that better affective team functioning increases openness to new practices but has less impact on implementation processes and outcomes. These findings are consistent with theory and empirical evidence that affective functioning is associated with workplace innovation [38, 70–73] and suggest that good affective functioning may be a necessary precondition for successful implementation, but insufficient on its own to improve implementation outcomes.

Behavioral and cognitive team functioning were correlated with implementation climate, but not significantly associated with any outcomes in multivariable models. These findings are surprising given substantial evidence that they are associated with innovation and performance in teams in other settings [38, 41]. It must be noted that dimensions of team functioning showed moderate to high multicollinearity, making it challenging to distinguish their unique contributions to implementation outcomes. Our measure of learning behavior was not specific to the CPM-PTS; it is possible that teams’ learning behavior did not extend equally to their use of the CPM-PTS. Measuring the extent to which team members sought feedback, shared information, and reflected on their use of the CPM-PTS specifically may have led to stronger associations with implementation outcomes. Similarly, our measure of clear direction assessed the extent to which the team shared broad goals, rather than goals specific to the CPM-PTS.

Better team performance was associated with more positive perceptions of the CPM-PTS and more positive implementation climate. It was not significantly associated with time to adoption or screening rates. These findings suggest that higher-performing teams may create better contexts for implementation, but this may not be sufficient to change behavior of those responsible for implementing new practices. Dimensions of teamwork may moderate the effects of implementation strategies or antecedents on implementation outcomes. Prior research in organizations has found that organizational climate moderates the effects of implementation climate, such that implementation climate is only associated with implementation outcomes in organizations with a more positive climate [74]. It is possible that similar associations exist at the team level, with team functioning and performance acting as moderators of the effect of implementation climate on outcomes. One study found that an innovation (i.e., electronic health record use) was associated with increased coordination and improved clinical outcomes only in highly cohesive teams [75, 76]. Longitudinal studies assessing both subjective and objective team performance in the context of implementation are needed to advance our understanding of how team performance affects implementation outcomes. Longitudinal studies could also test how team constructs affect the sustainability of innovations in team-based settings.

Strengths of this study include our assessment of multiple aspects of teamwork and use of both self-reported and administrative data to assess multiple implementation outcomes. The teams included in this study had fluid and dynamic membership, like many healthcare and human service teams, and their multidisciplinary cross-sector nature parallels that of many implementation teams, increasing the generalizability of these findings. Key limitations of the study include its cross-sectional nature, variability in the length of time between initial implementation and the team survey, the small number of teams, and shared method variance, as most measures were rated by team members. Although shared method variance could inflate associations, especially between team constructs and individual-level implementation outcomes, we found different patterns of associations between team constructs and implementation outcomes, suggesting this issue had minimal impact on our findings. We collected data from all CACs participating in this statewide implementation effort and had excellent participation from team members. However, because survey invitations were sent via directors, it is possible that some team members were not invited to participate. In addition, the small number of teams provided limited power to detect center-level associations and precluded testing more complex associations between team constructs, including moderation or mediation.

Teams are complex, adaptive, dynamic systems, and processes, and performance are likely to influence one another over time [77, 78]. Research with larger samples of teams could allow for the exploration of mediational pathways (e.g., interdependence to functioning to outcomes; functioning to implementation climate to outcomes) and potential moderators. It is possible that aspects of team functioning moderate one another; for instance, learning behavior may only improve implementation outcomes when teams share clear goals. Qualitative studies describing team members’ involvement in implementation and mixed methods approaches can also inform our understanding of team-level influences on implementation and may help identify team-level mechanisms of change in multidisciplinary team-based service settings.

## Conclusions

Team-based care is increasingly common in healthcare and human service settings, yet little research has examined how teamwork may influence implementation processes and outcomes. In our study of cross-sector multidisciplinary teams implementing a mental health screening/referral protocol, we found that aspects of team interdependence, functioning, and performance were associated with individual- and center-level implementation outcomes. Greater understanding of how teamwork affects implementation can facilitate the development of implementation strategies to improve teams’ capacity to implement evidence-based practices and enhance the quality of care in team-based settings. 

## Supplementary Information

**Additional file 1: Table S1.** Descriptive Statistics and Correlations among Individual-Level Measures (*N* = 384-426)

**Additional file 2: Fig. S1.** Task Interdependence and Center-level Implementation Outcomes. **Fig. S2.** Outcome Interdependence and Center-level Implementation Outcomes. **Fig. S3.** Affective Integration and Center-level Implementation Outcomes. **Fig. S4.** Learning Behavior and Center-level Implementation Outcomes. **Fig. S5.** Clear Direction and Center-level Implementation Outcomes. **Fig. S6.** Team Performance and Center-level Implementation Outcomes.

## Data Availability

The datasets analyzed during the current study are available from the corresponding author upon reasonable request.

## Footnotes

1 The team with 110 members was much larger than all other teams, in part because this urban CAC handled cases from many law enforcement jurisdictions. With this team excluded, teams ranged in size from 9 to 48 members (*M* = 25; SD = 12; interquartile range [IQR] = 15–36). Team size remained significantly correlated with participation rate (*r* = − .47, *p* < .05). The pattern of results did not change when this team was excluded from the analyses.
