# Supplementary material for: Associations between teamwork and implementation outcomes in multidisciplinary cross-sector teams implementing a mental health screening and referral protocol
Source: Implement Sci Commun. 2023 Feb 10;4:13. doi: 10.1186/s43058-023-00393-8 (PMC9921625; doi:10.1186/s43058-023-00393-8)
Supplement: Supplementary file 1 — Additional file 1: Table S1. Descriptive Statistics and Correlations among Individual-Level Measures (N = 384-426) [file 43058_2023_393_MOESM1_ESM.docx]

**Additional File 1**

**Additional Table 1**

*Descriptive Statistics and Correlations among Individual-Level Measures (N = 384-426)*

|  | **M (SD)** | **Range** | **2** | **3** | **4** | **5** | **6** | **7** | **8** | **9** |
| --- | --- | --- | --- | --- | --- | --- | --- | --- | --- | --- |
| **1.** Task Interdependence | 3.84 (.72) | 1.00-5.00 | .23** | .21** | .18** | .12* | .09 | .09 | .06 | .08 |
| **2.** Outcome Interdependence | 3.66 (.85) | 1.00-5.00 |  | .46** | .59** | .68** | .48** | .04 | .05 | .06 |
| **3.** Affective Integration | 4.28 (.55) | 2.36-5.00 |  |  | .56** | .55** | .62** | .19** | .17** | .18** |
| **4.** Learning Behavior | 4.54 (.95) | 1.29-6.71 |  |  |  | .71** | .53** | .10* | .08 | .11* |
| **5.** Clear Direction | 5.41 (1.22) | 1.00-7.00 |  |  |  |  | .57** | .08 | .09 | .08 |
| **6.** Team Member-rated Performance | 5.78 (.91) | 2.60-7.00 |  |  |  |  |  | .13* | .12* | .11* |
| **7.** Acceptability | 4.05 (.73) | 1.00-5.00 |  |  |  |  |  |  | .85** | .86** |
| **8.** Appropriateness | 4.14 (.75) | 1.00-5.00 |  |  |  |  |  |  |  | .91** |
| **9.** Feasibility | 4.06 (.74) | 1.00-5.00 |  |  |  |  |  |  |  |  |

** *p* < .01 * *p* < .05
