# Supplementary material for: Associations between teamwork and implementation outcomes in multidisciplinary cross-sector teams implementing a mental health screening and referral protocol
Source: Implement Sci Commun. 2023 Feb 10;4:13. doi: 10.1186/s43058-023-00393-8 (PMC9921625; doi:10.1186/s43058-023-00393-8)

**Additional File 2**

Figure 1: Task Interdependence and Center-level Implementation Outcomes

Figure 2: Outcome Interdependence and Center-level Implementation Outcomes

Figure 3: Affective Integration and Center-level Implementation Outcomes

Figure 4: Learning Behavior and Center-level Implementation Outcomes

Figure 5: Clear Direction and Center-level Implementation Outcomes

Figure 6: Team Performance and Center-level Implementation Outcomes

**Figure 1**

*Task Interdependence and Center-level Implementation Outcomes*


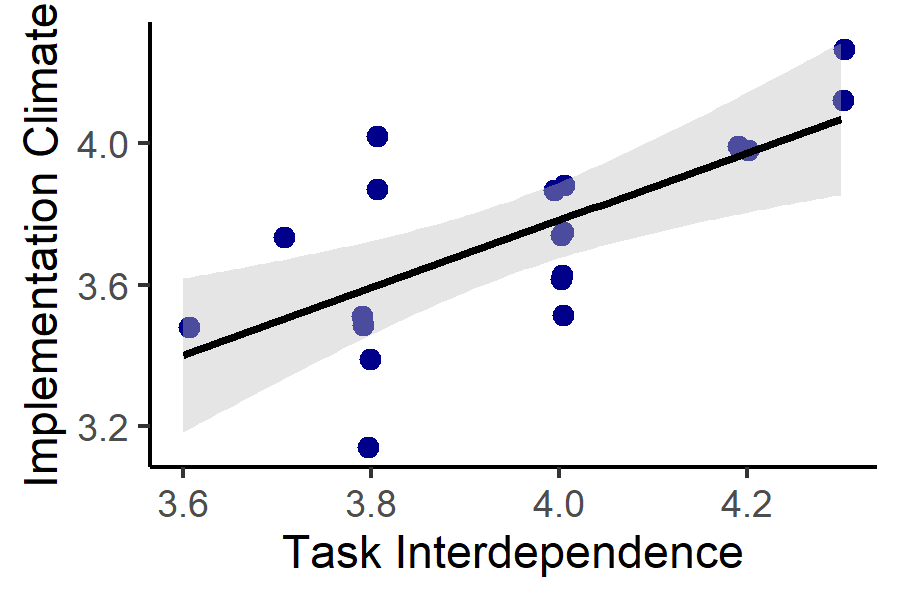

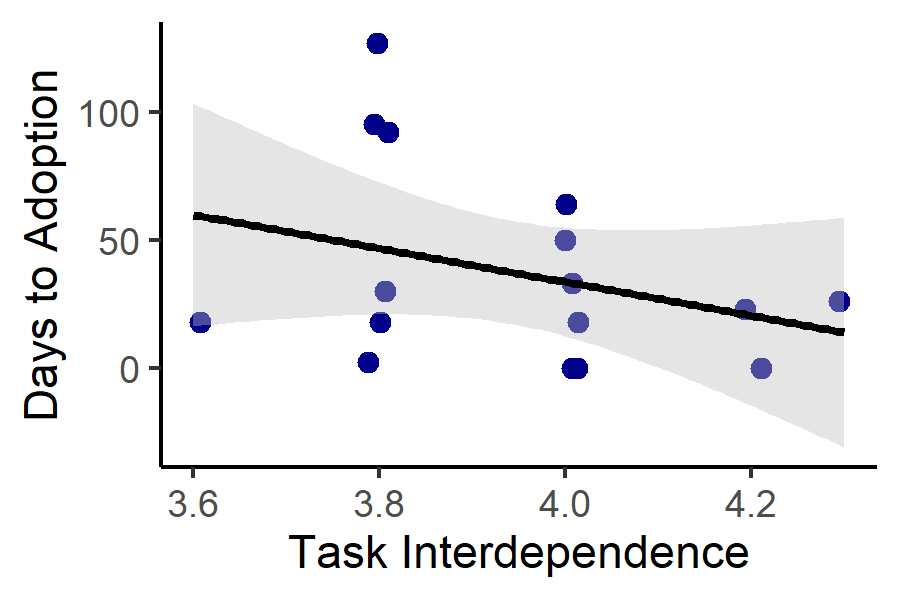


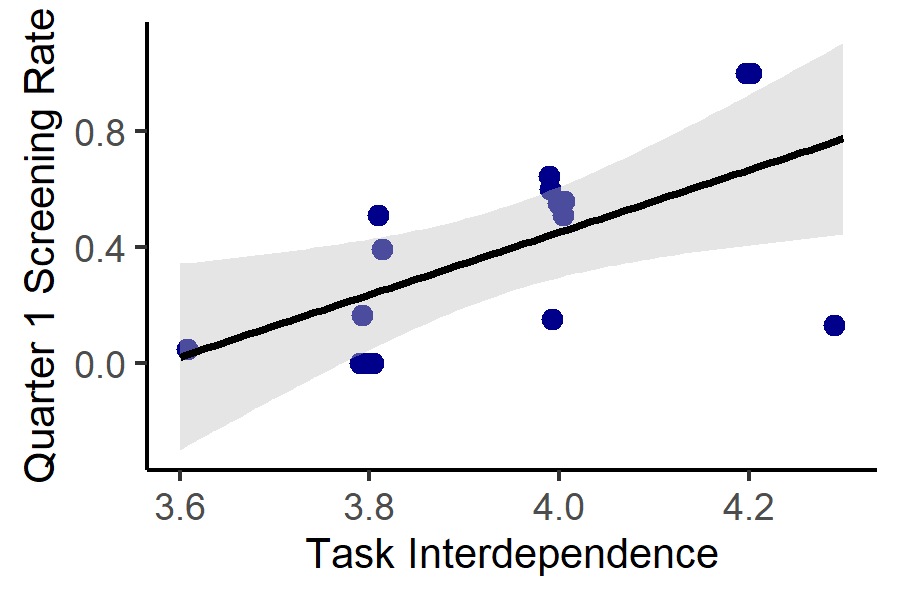

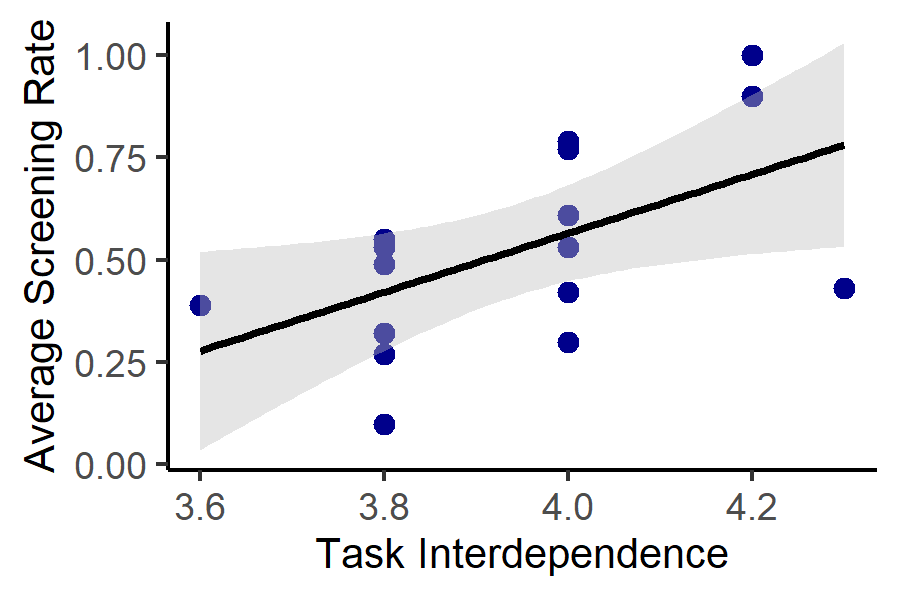


**Figure 2**

*Outcome Interdependence and Center-level Implementation Outcomes*


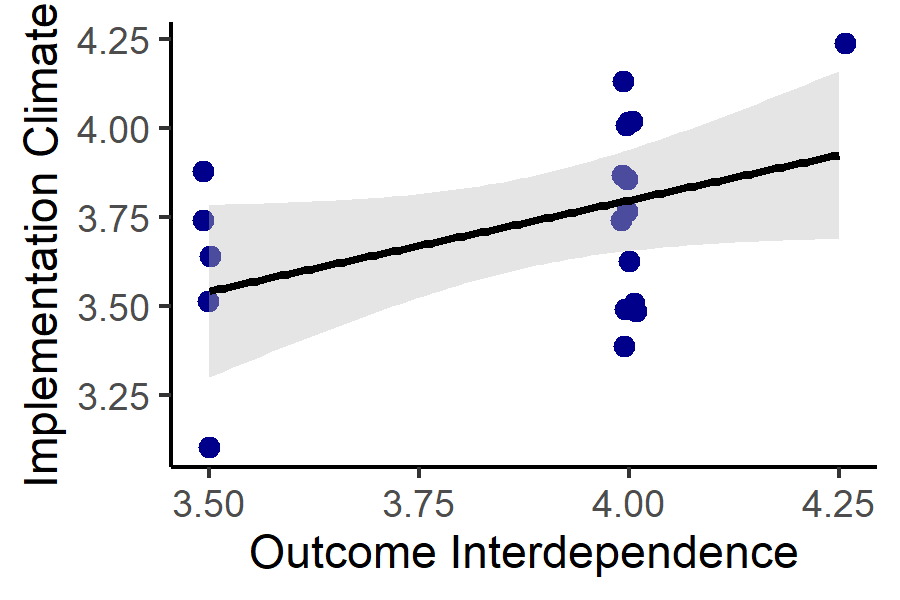

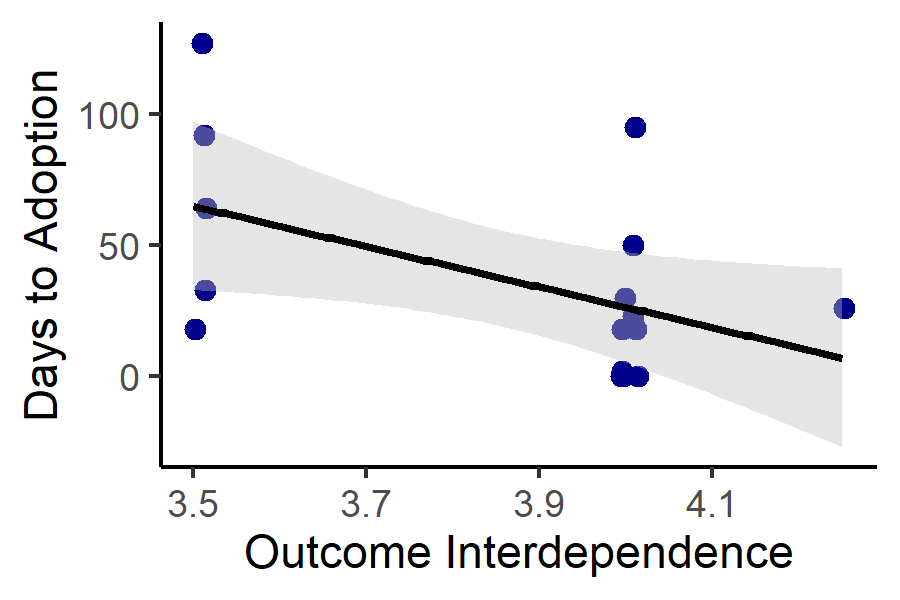


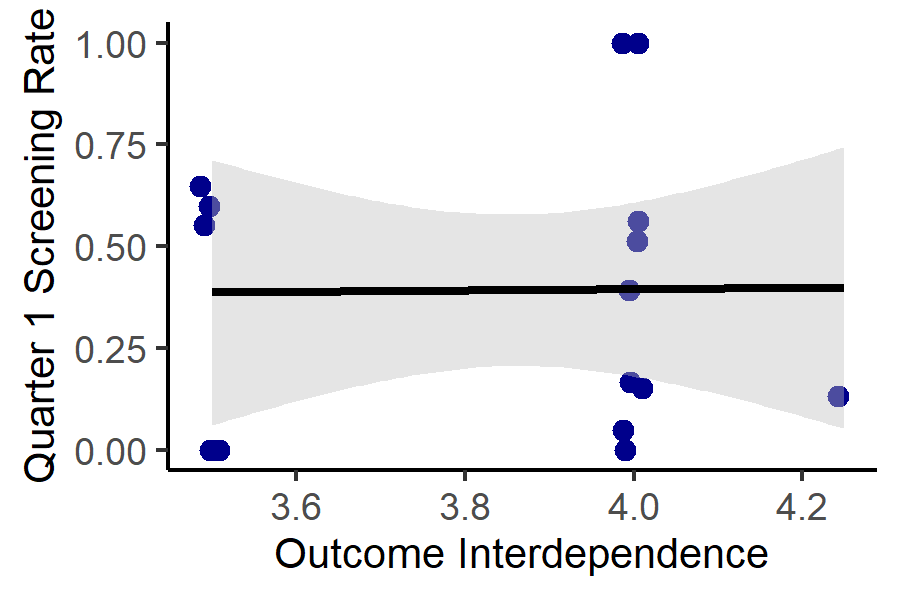

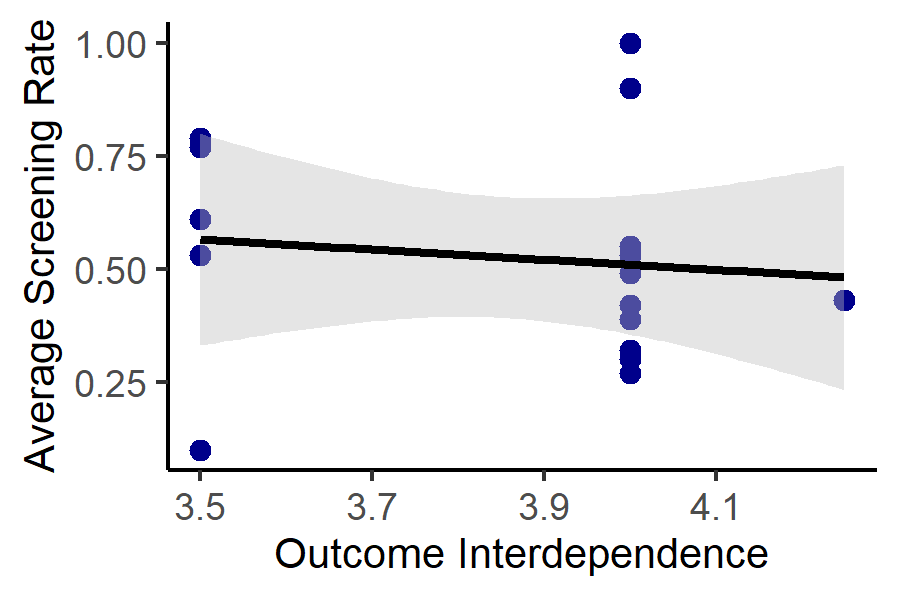


**Figure 3**

*Affective Integration and Center-level Implementation Outcomes*


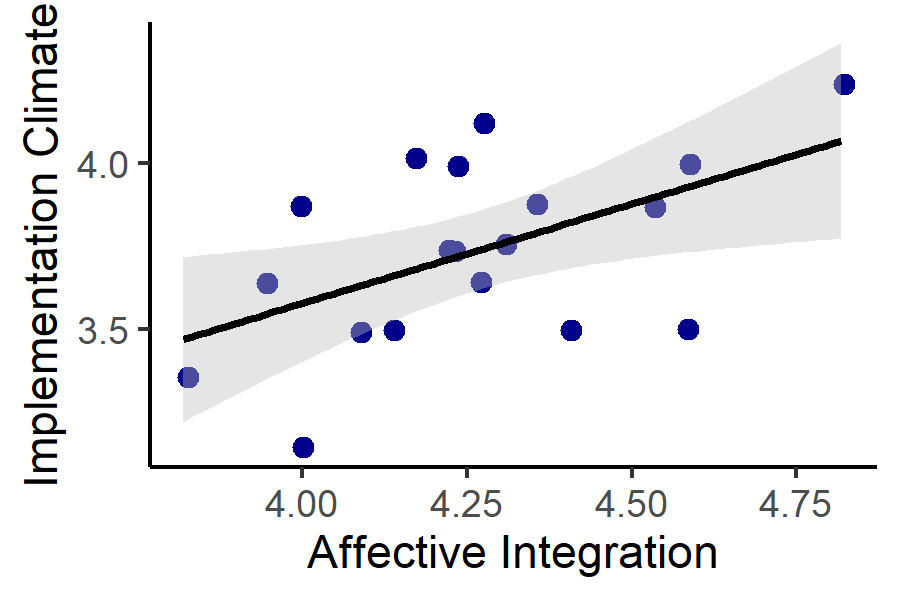

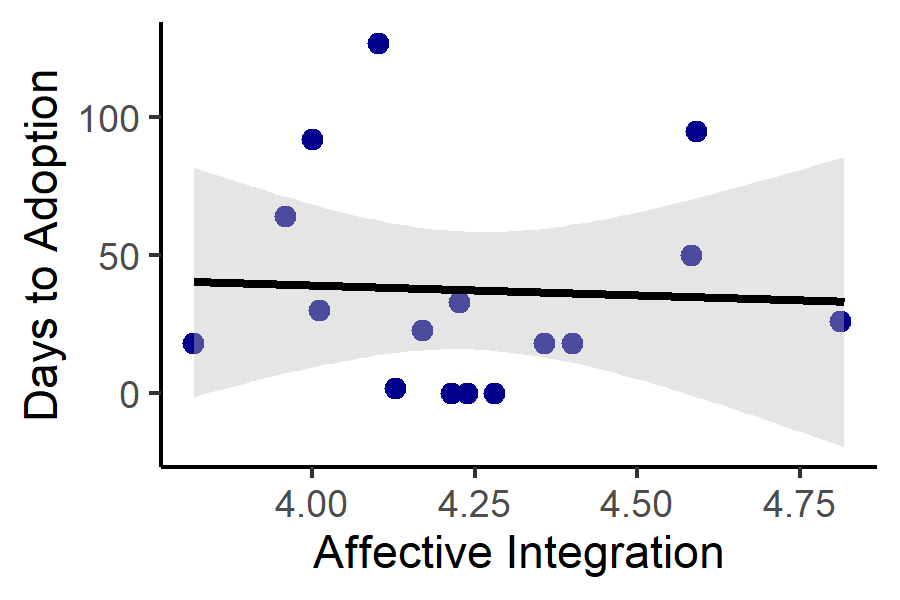


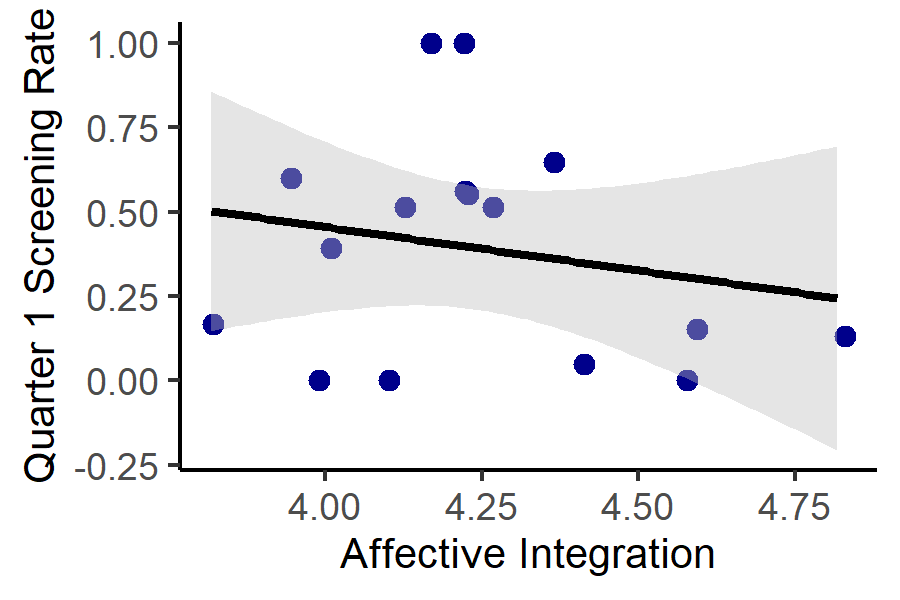

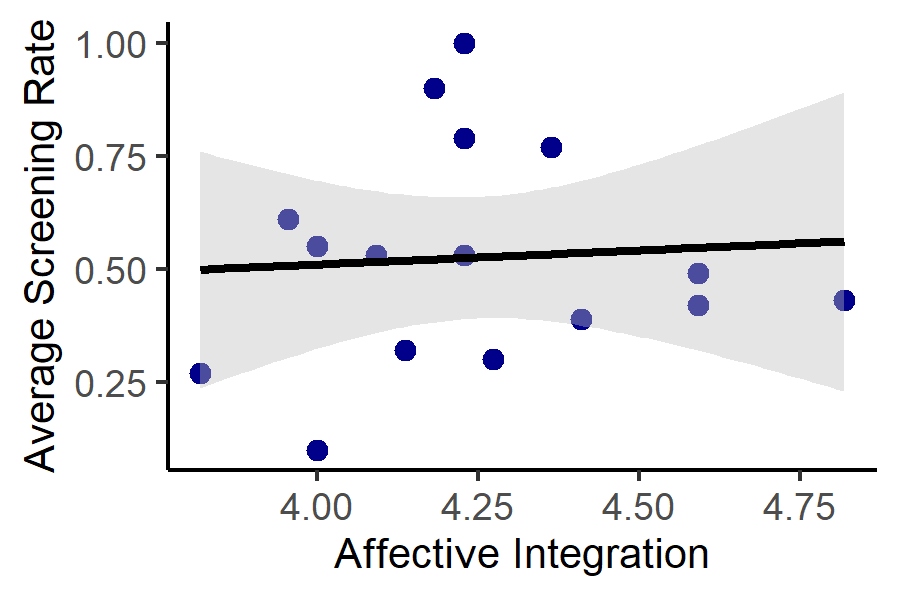


**Figure 4**

*Learning Behavior and Center-level Implementation Outcomes*


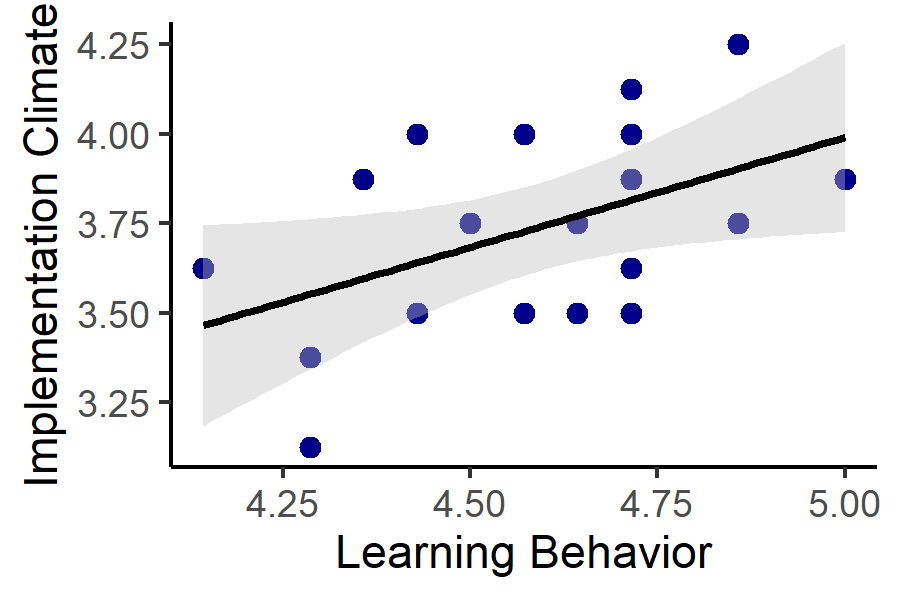

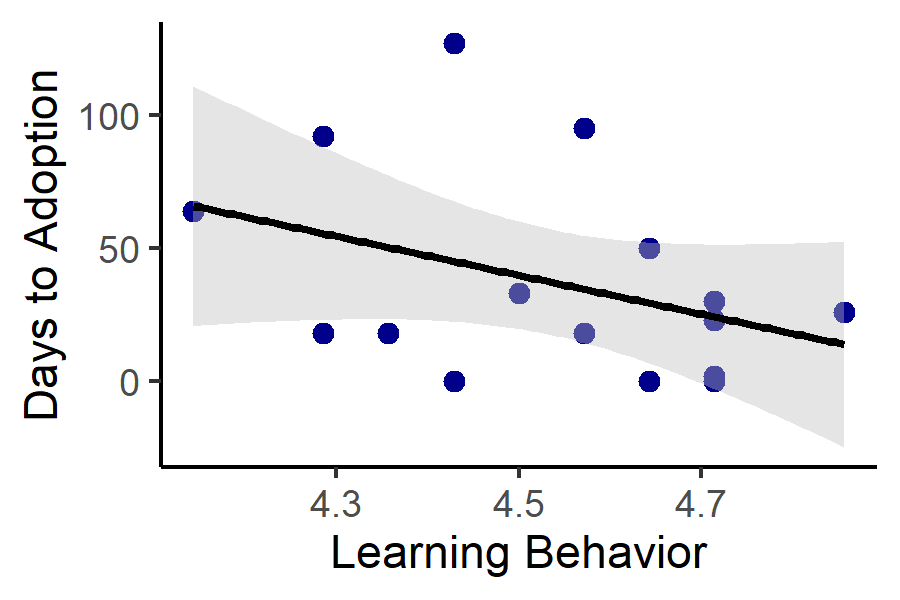


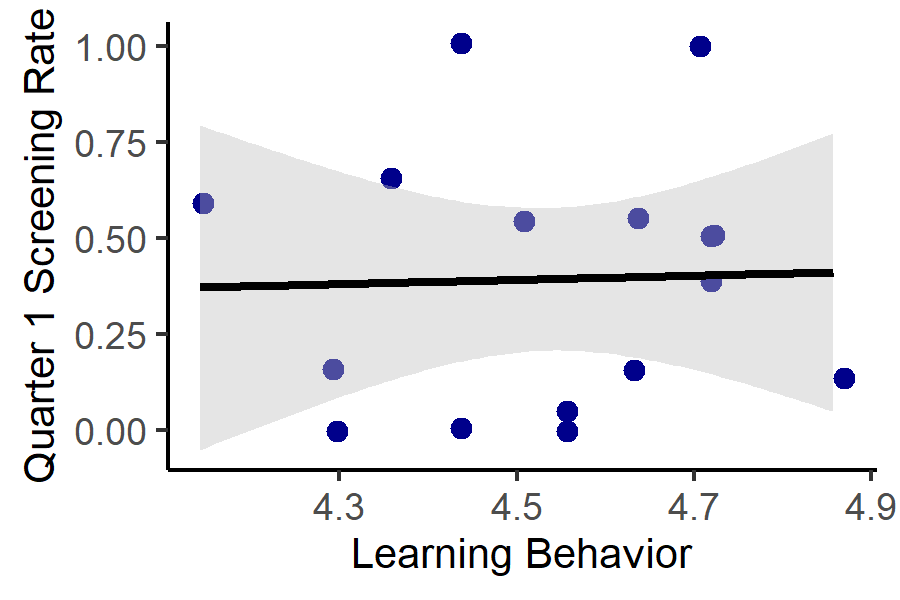

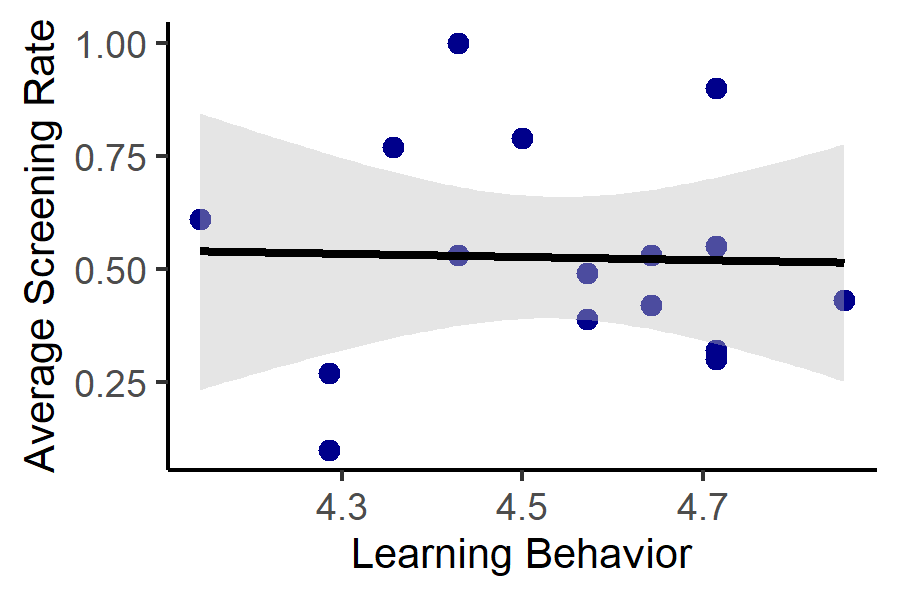


**Figure 5**

*Clear Direction and Center-level Implementation Outcomes*


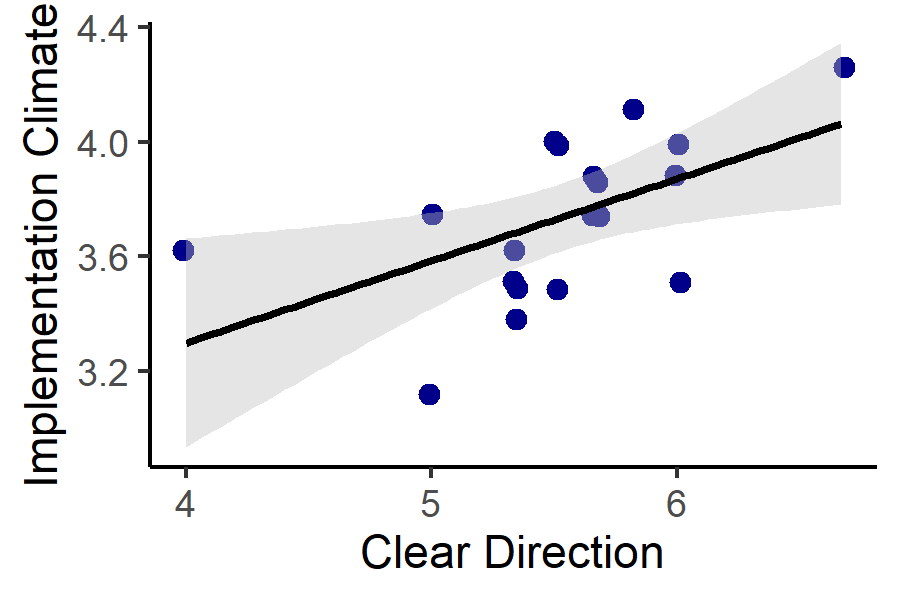

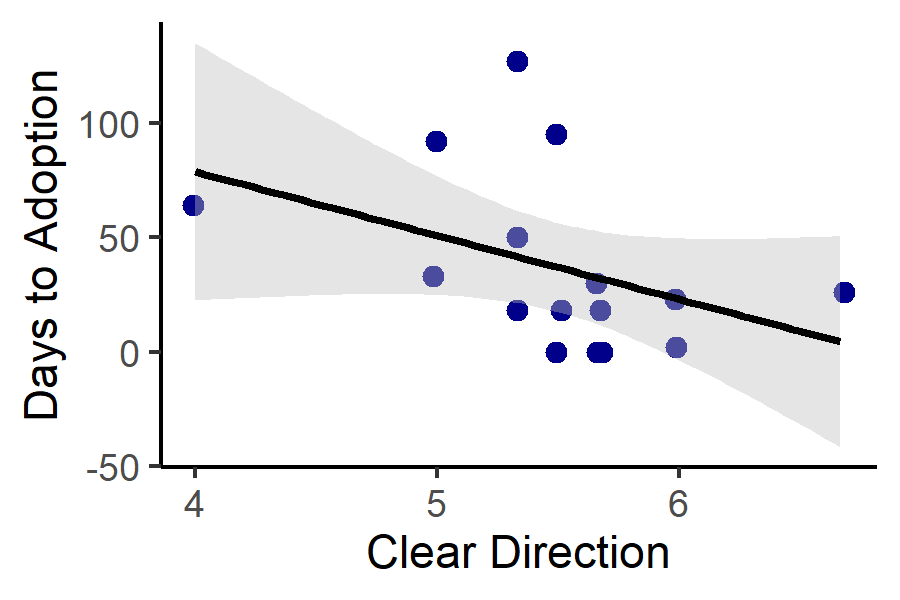


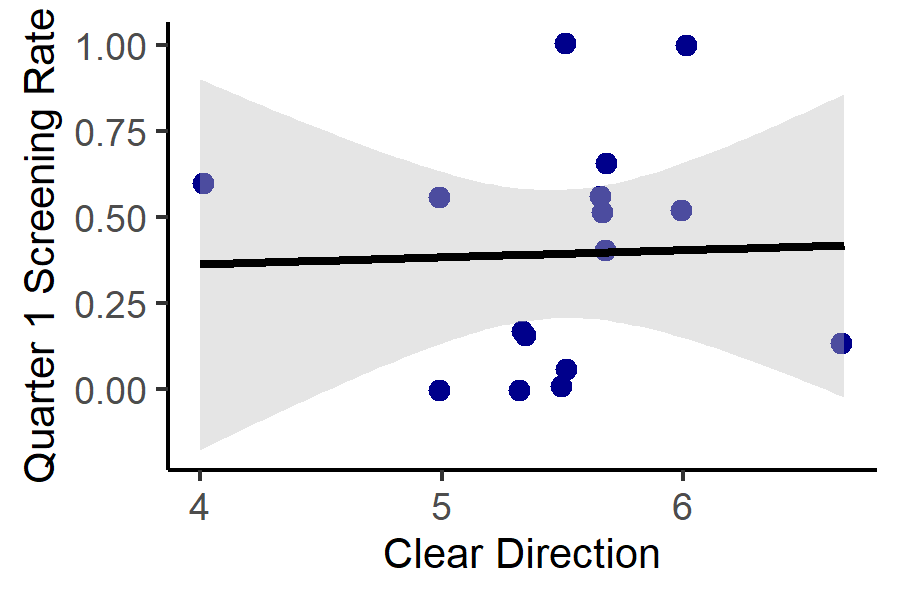

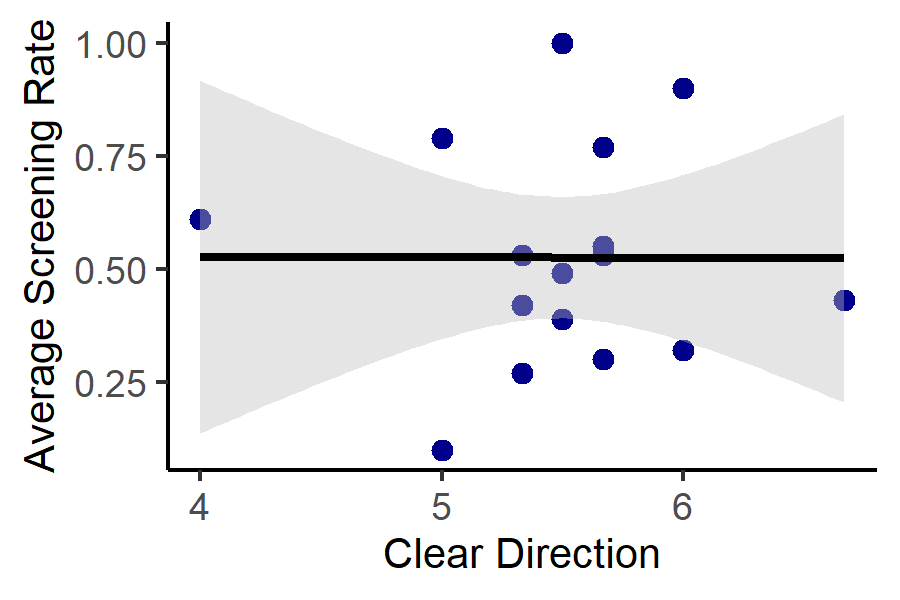


**Figure 6**

*Team Performance and Center-level Implementation Outcomes*


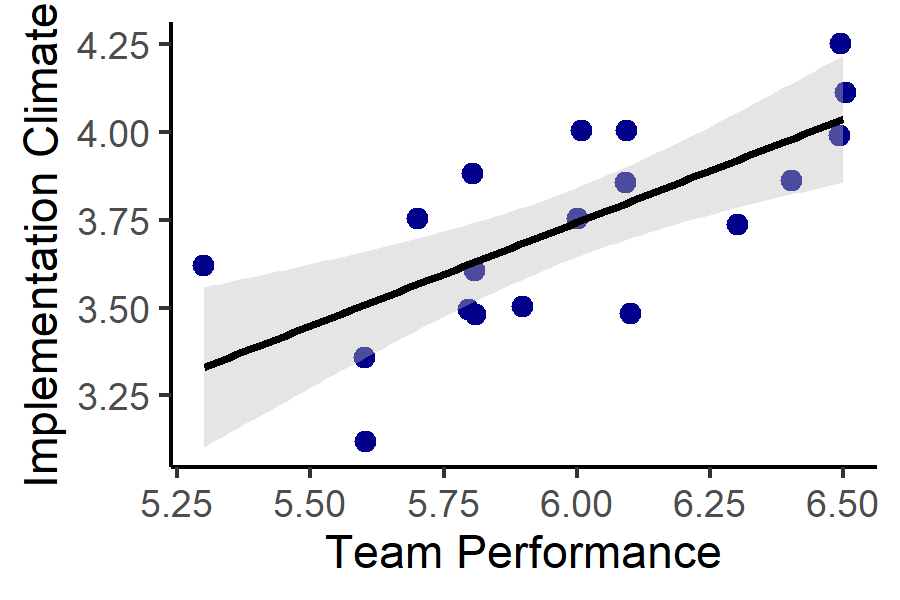

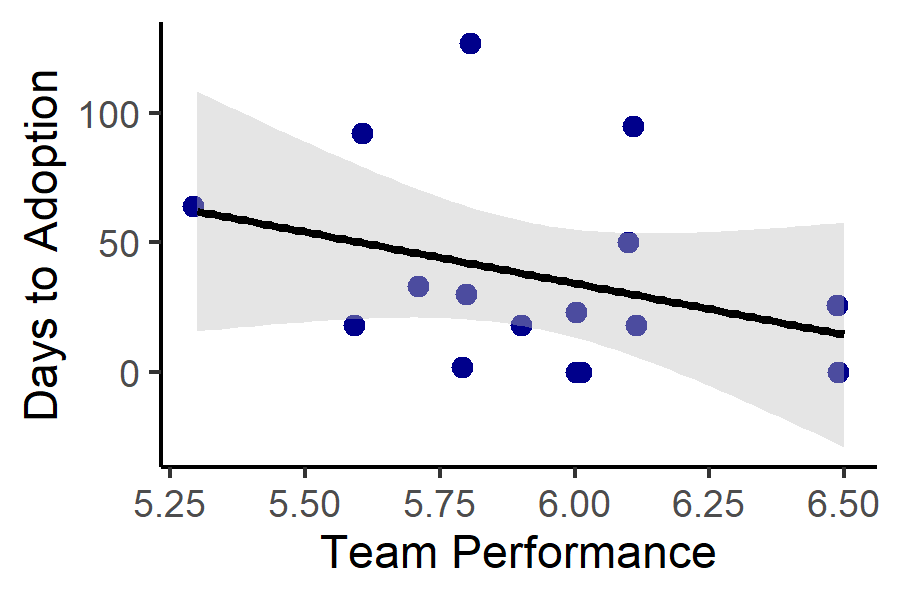


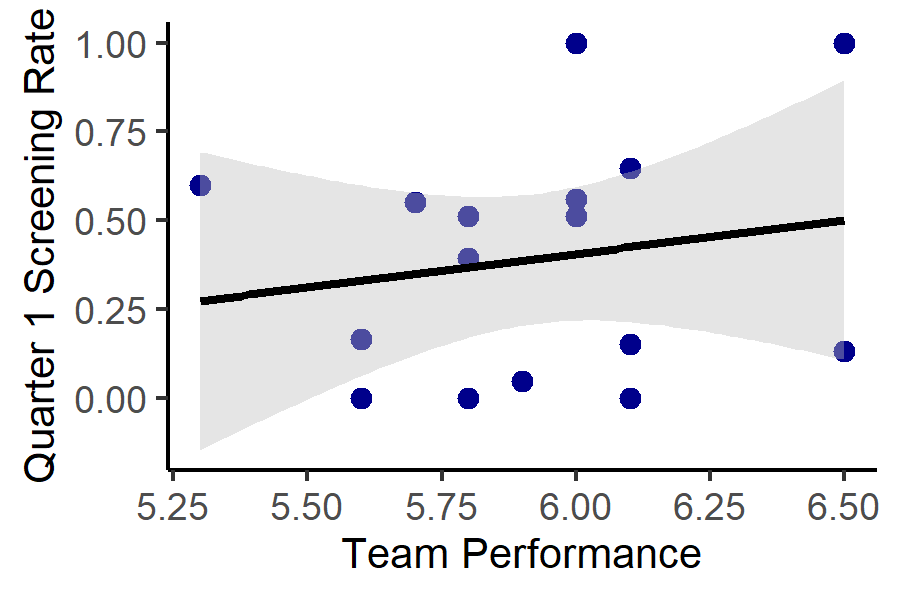

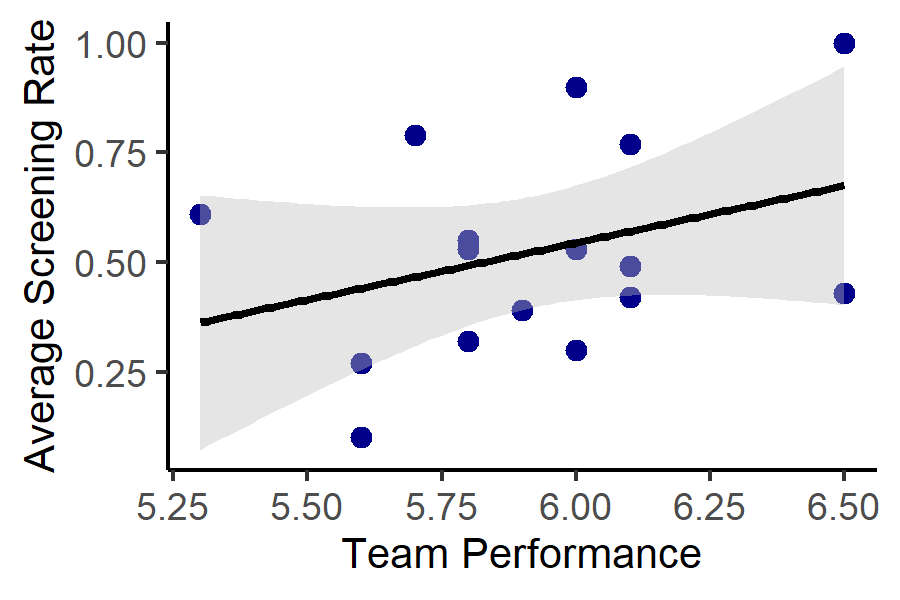

Supplement: Supplementary file 2 — Additional file 2: Fig. S1. Task Interdependence and Center-level Implementation Outcomes. Fig. S2. Outcome Interdependence and Center-level Implementation Outcomes. Fig. S3. Affective Integration and Center-level Implementation Outcomes. Fig. S4. Learning Behavior and Center-level Implementation Outcomes. Fig. S5. Clear Direction and Center-level Implementation Outcomes. Fig. S6. Team Performance and Center-level Implementation Outcomes. [file 43058_2023_393_MOESM2_ESM.docx]
